# Detection of Food Spoilage and Pathogenic Bacteria Based on Ligation Detection Reaction Coupled to Flow-Through Hybridization on Membranes

**DOI:** 10.1155/2014/156323

**Published:** 2014-04-10

**Authors:** K. Böhme, P. Cremonesi, M. Severgnini, Tomás G. Villa, I. C. Fernández-No, J. Barros-Velázquez, B. Castiglioni, P. Calo-Mata

**Affiliations:** ^1^Department of Analytical Chemistry, Nutrition and Food Science, School of Veterinary Sciences, University of Santiago de Compostela, 27002 Lugo, Spain; ^2^Institute of Agricultural Biology and Biotechnology, National Research Council, 26500 Lodi, Italy; ^3^Institute of Biomedical Technologies, Italian National Research Council, 20090 Segrate, Italy

## Abstract

Traditional culturing methods are still commonly applied for bacterial identification in the food control sector, despite being time and labor intensive. Microarray technologies represent an interesting alternative. However, they require higher costs and technical expertise, making them still inappropriate for microbial routine analysis. The present study describes the development of an efficient method for bacterial identification based on flow-through reverse dot-blot (FT-RDB) hybridization on membranes, coupled to the high specific ligation detection reaction (LDR). First, the methodology was optimized by testing different types of ligase enzymes, labeling, and membranes. Furthermore, specific oligonucleotide probes were designed based on the 16S rRNA gene, using the bioinformatic tool Oligonucleotide Retrieving for Molecular Applications (ORMA). Four probes were selected and synthesized, being specific for *Aeromonas* spp., *Pseudomonas* spp., *Shewanella* spp., and *Morganella morganii*, respectively. For the validation of the probes, 16 reference strains from type culture collections were tested by LDR and FT-RDB hybridization using universal arrays spotted onto membranes. In conclusion, the described methodology could be applied for the rapid, accurate, and cost-effective identification of bacterial species, exhibiting special relevance in food safety and quality.

## 1. Introduction 

Microbial spoilage of food is an area of global concern, causing serious foodborne intoxications and resulting in high economic losses for the food producing sector. The microbiological safety of food has become an increased importance for the consumers, as well as for the food producing industry, and regulations that require monitoring and the enforcement of control systems have been established. Research in the food safety and quality field has been continuously focused on the search for sensitive, accurate, rapid, and cost-effective methods to determine potential microbial risks. Conventional cultivation methods and phenotypical tests are still commonly applied in the food microbiological field but are time consuming and labor intensive. Over the past years, the tools for molecular diagnosis have greatly improved and immunoassays, mass spectrometry, and PCR-based technologies have been introduced for microbial identification. Microarrays are powerful, sensitive, and specific high-throughput technologies that allow an accurate identification based on single target detection and can determine subtle differences in the bacterial genomes. Gene arrays can hybridize multiple DNA targets simultaneously and thus have enormous potential for the detection and identification of bacterial species [1]. Thanks to the increase in the complete microbial genome sequences, DNA microarrays are becoming a common tool in many areas of microbial research [2]. For bacterial identification, the so-called oligonucleotide arrays are used, consisting of short oligonucleotide probes, specific for the corresponding target sequence. In the last decade, oligonucleotide arrays have been successfully applied for the detection of bacterial pathogens in the clinical, as well as in the food sector [3–7]. However, besides numerous advantages, such as being informative and highly repeatable and the potential for miniaturization and ease of automation, most described microarray methods require elevated costs for equipment and a more qualified personal, circumstances that limit its broad application for routine analyses in common food control laboratories. In this sense, reverse dot-blot (RDB) hybridization using membrane-based macroarrays represents a cost-effective alternative that needs less specialized equipment and can be used in any well set up molecular biology laboratory [8]. Moreover, a hybridization system in which, with the help of negative pressure, DNA samples actively flow through membranes and the immobilized probes have been developed. This flow-through hybridization system is the most efficient method for molecular hybridization and has the advantages of being very fast, semiautomated, clean, versatile, and less expensive than traditional hybridization. In addition, the diffusivity and local reaction concentration of DNA are increased due to three-dimensional volumes, leading to a higher sensitivity [9].

Although the basic principle of the hybridization technique is the discrimination between a perfect match and one single mismatch, this specificity is difficult to reach with classical oligoprobe arrays and cross-reactions can lead to false positive results [10]. This is of special relevance to the food control sector, since bacterial species present in food products can differ significantly in their pathogenic and spoilage nature and it is necessary to detect specifically those that represent a high microbial risk. With the ligase detection reaction (LDR), sequences can be differentiated by just one polymorphism and closely related bacterial species with high sequence similarity can be discriminated. The microarray technology based on LDR coupled to a universal oligonucleotide array has been described elsewhere [11] and requires the design of two oligonucleotide probes specific for each target sequence. The discriminating probe carries a label at the 5′ end and the discriminating base at the 3′ end. The common probe contains a unique sequence at the 3′ end that is addressed to a certain location on a universal array (zipcode). In the ligase reaction both probes are joined, only in the case that a complementary template is present and this template sequence exhibits a perfect match in the discriminating position. The described methodology has been successfully applied for the detection and identification of pathogenic bacterial species in milk [11].

Based on these previous works [11], the present work focused on the design of specific LDR-probes for the detection of food spoilage bacterial species. Accordingly, 16S rRNA gene sequences of the most relevant foodborne pathogenic and spoilage bacterial species were considered and single nucleotide polymorphisms for the discrimination at the species level were determined with the bioinformatics tool ORMA [12].

The objective of this study was the development of a fast and cost-effective flow-through hybridization technology, based on the high specific LDR approach coupled to universal macroarrays on membranes. The proposed methodology combines the efficiency and specificity of the microarray technique with the rapidity and convenience of flow-through hybridization, being of special interest for microbial identification in the food safety and quality field. The application of this sensitive method would result in the rapid and early detection of food spoilage bacteria and thus contribute to the accurate analysis and control of microbial risks.

## 2. Materials and Methods

### 2.1. Design of Specific Oligonucleotide Probes

For the probe design, up to fifty 16S rRNA gene sequences of bacterial species exhibiting pathogenic or spoilage activity were downloaded from public databases (NCBI, RDP) and aligned with the bioinformatics program ClustalX 2.1 [13]. Consensus sequences were determined for every species and/or genus with the program GenDoc 2.7 [14], considering polymorphisms with a percentage higher than 30%. Based on the consensus sequences of every species and/or genus, candidate probes were designed with the bioinformatics tool Oligonucleotide Retrieving for Molecular Applications (ORMA) [12]. All potential candidate probes were tested* in silico* against reference databases (RDP, NCBI) for their specificity. At the end four probes were selected to carry out the studies of the present work, being specific for the genera* Aeromonas*,* Pseudomonas*,* Shewanella*, and for the species* Morganella morganii*, respectively. The sequences of the specific probe pairs and the corresponding zipcode positions are listed in Table 1. The DNA sequences of the zipcodes were randomly selected as previously described [15].

### 2.2. Bacterial Strains and Culture Media

For the validation of the synthesized probes 16 reference strains were tested (Table 2). Bacterial reference strains were obtained from the Spanish Type Culture Collection (CECT). The strains were reactivated in brain-heart infusion (BHI) broth (Becton, Dickinson and Company, Le Pont de Claix, France) and incubated for 24 h at 30°C. Afterwards, bacterial cultures were grown on plate count agar (PCA) (Oxoid, Hampshire, England) at 30°C and single colonies were isolated. For DNA extraction a single colony was incubated in BHI for 24 h at 30°C.

### 2.3. DNA Extraction and PCR Amplification

Total genomic DNA was extracted by means of the DNeasy tissue minikit (Qiagen, Valencia, CA, USA) and the 16S rRNA gene was amplified as described elsewhere [11], using the universal primer pair 20F/1500R [16]. The PCR products were purified by using a Wizard SV gel and PCR Clean-Up System purification kit (Promega Italia, Milan, Italy) and quantified by electrophoresis in agarose gel with the quantitative LowRanger 100 bp DNA Ladder (Norgen Biotek Corp., Thorold, Ontario, Canada).

### 2.4. Ligase Detection Reaction (LDR)

Selected probes were ordered for synthesis (Thermo Fisher Scientific GmbH, Ulm, Germany). Each probe pair consisted of a common probe with a phosphate modification at the 5′ end and a unique sequence (complementary zipcode) at the 3′ end and a discriminating probe with the specific nucleotide at the 3′ end and a label for detection at the 5′ end. Three different labels were tested: Cy3, Cy5, and biotin.

The lyophilized probes were resuspended in water, obtaining a concentration of 100 *μ*M. Afterwards, a probe mix was prepared, containing 1 *μ*M of every oligonucleotide of each probe pair. A probe pair complementary to a synthetic DNA template was also added as a ligation control to the probe mix (see Table 1). The total volume of LDR was 20 *μ*L and it included 1X ligase buffer, 4 U ligase, 0.05 *μ*M of each probe, 0.5 *μ*M synthetic DNA template, and 5–50 fmol of the PCR products. Three ligase enzymes were tested:* pfu* DNA ligase (Stratagene, La Jolla, CA, USA), ampligase (Epicentre, Madison, WI, USA), and* taq* ligase (New England Biolabs, Beverly, MA, USA).

### 2.5. Denaturation of LDR Products

LDR hybridization mixtures of a total volume of 65 *μ*L were prepared, containing 20 *μ*L of the LDR products, 0.1 *μ*M labeled* c*zipcode 66 (complementary to zipcode 66, see Table 1), 0.1 mg/mL salmon sperm, and Solution A (2X SSC/0.1% SDS). Before hybridization to the universal arrays or membranes was performed, these mixtures were denatured at 95°C for 2 min and chilled on ice.

### 2.6. Membrane Preparation and Subsequent Immobilization of Oligonucleotides

The amino-modified zipcode oligonucleotides (100 *μ*M; Thermo Fisher Scientific GmbH) were diluted with 0.5 M sodium bicarbonate buffer (pH 8.4) to different concentrations (1 *μ*M, 0.1 *μ*M, and 0.01 *μ*M) and were dotted at different volumes (1 *μ*L, 0.5 *μ*L, 0.3 *μ*L, 0.2 *μ*L, and 0.1 *μ*L) at the given positions on the membranes.

One membrane contained nine subarrays, allowing the hybridization of nine samples simultaneously. Two different types of membranes were tested and required different preparation procedures. Nylon Biodyne C membranes (Negatively-Charged Nylon 66; pore size: 0.45 *μ*m; Pall Co.) were rinsed briefly with 0.1 M HCl and then incubated in freshly prepared 16% EDC (N-(3-dimethylaminopropyl)-N-ethylcarbodiimide hydrochloride; Sigma-Aldrich) for 15 min. After drying at air and dotting the oligonucleotides, any remaining active groups were quenched with 0.1 M NaOH for 10 min. Finally, membranes were rinsed thrice with deionized water and air-dried for storage or immediately used for hybridization.

The Protran BA 85 nitrocellulose membranes (pore size: 0.45 *μ*m; Sigma-Aldrich) were used without any pretreatment. After dotting the oligonucleotides, membranes were exposed to UV-light (254 nm) for 30 sec to cross-link the oligonucleotides.

### 2.7. Flow-Through Hybridization on Membranes

Flow-through hybridization was performed on an HybriMax device (Patent no. US6020187; Hybribio Limited, Hong Kong, China) [17] that works on the basis of the particular principle of flow-through hybridization caused by a negative pressure under the airproof hybridization membrane produced by pumping. The steps were as follows: (1) prehybridization of the membrane with 0.5 mL Solution A, (2) dilution of the denatured LDR products with 450 *μ*L Solution A and hybridization to the membrane at 50°C for 15 min, and (3) washing the membrane twice with 0.5 mL Solution A to eliminate the unhybridized PCR products.

When working with biotin-labeled probes, color development was carried out as follows: (4) washing the membrane twice with 0.5 mL 0.1 M sodium citrate, (5) incubation with 0.5 mL streptavidin-horseradish peroxidase (HRP) at 37°C for 5 min, (6) washing the membrane twice with 0.5 mL Solution A to eliminate the uncombined peroxidase, (7) washing the membrane twice with 0.5 mL 0.1 M citric acid buffer (pH 5), and (8) color development with 0.5 mL 3,3,5,5-tetramethylbenzidine (TMB) chromogen.

The membranes were imaged and analyzed with the ChemiDoc MP imaging system (BioRad).

### 2.8. Validation of Probes by Hybridization to an Universal Array (UA) on Glass Slides

At the same time, the probes were validated by hybridization of the LDR reaction products to a universal microarray on glass slides, following the methodology described elsewhere [11]. One slide contained eight subarrays, separated by press-to-seal silicone isolators (1.0 × 9 mm; Schleicher and Schuell BioScience, Dassel, Germany), allowing the hybridization of eight samples simultaneously. Briefly, the denatured LDR products were applied to the slide and hybridization was carried out in a dark chamber at 65°C for 1 h 30 min. After hybridization, the slides were washed at 65°C for 15 min in Solution A. Finally, the slide was dried by spinning at 80 ×g for 3 min. The fluorescent signals were acquired at a 5 *μ*m resolution by using a ScanArray Lite laser scanning system (Perkin Elmer Life and Analytical Sciences, Boston, MA) with a green laser for Cy3 dye (*λ*_ex_, 543 nm; *λ*_em_, 570 nm). All images were analyzed visually and statistically, determining average values and standard deviations of the fluorescence intensity.

## 3. Results

### 3.1. Probe Validation by Hybridization to Universal Arrays on Glass Slides

For the probe design, we considered DNA sequences of the most important food spoilage and pathogenic bacterial species, using the 16S rRNA gene as target. Consensus sequences were determined for every species and/or genus of interest and discriminating nucleotides were determined with the computer software ORMA. The list of specific fragments of 25–60 bp was reviewed and the potential candidate probes were tested* in silico* against reference databases (RDP, NCBI) with the aim to verify their specificity. Four of the designed probes were selected for the present studies, for being specific for the bacterial species* M. morganii* and for* Aeromonas*,* Pseudomonas*, and* Shewanella* genera, which are of special interest in seafood spoilage.

Before applying the synthesized probes in the flow-through hybridization approach, they were tested* in vitro* by hybridization to universal arrays (UA) on glass slides, as described elsewhere [11]. The designed probes were specific for the corresponding species and/or genus and sensitive for concentrations between 5 and 50 fmol of sample DNA. Furthermore, the amplified 16S rDNAs of two additional reference strains (*Bacillus cereus* ATCC 14893 and* Staphylococcus aureus* ATCC 9144) were analyzed as negative controls, resulting in no fluorescence signal against any of the tested probes.

### 3.2. Optimization of the Flow-Through Hybridization Approach Coupled to LDR

The principle of the flow-through hybridization approach is based on the methodology of reverse dot blot (RDB) hybridization in that the targeting molecules flow through the immobilized probes within the membrane fibers. This reduces the time needed for hybridization from hours to minutes and also simplifies the washing steps, since any unbound molecule is removed easily by passing through the membrane.

**Figure 1:**
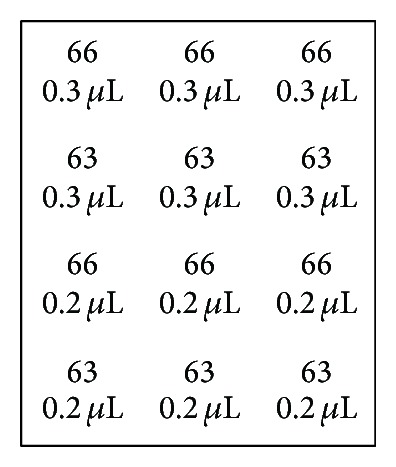
Scheme of spotted zipcode-oligonucleotides on membranes for the optimization experiments. ^66^Hybridization control; ^63^ligation control.

In the present study, we applied for the first time the LDR-based methodology to a flow-through hybridization strategy. First, with the aim of optimizing the protocol, a number of hybridizations were carried out under different conditions only with the positive controls for hybridization (66) and ligation (63). The hybridization control is a labeled synthetic oligonucleotide that contains a DNA sequence that is complementary to the zipcode 66 spotted onto the membrane: a positive signal at this position indicates that the hybridization process is successful. The ligation control includes a synthetic oligonucleotide and a probe pair that is complementary to the DNA sequence of the synthetic template. This probe also contains a DNA sequence that is complementary to the zipcode 63 spotted onto the membrane: a positive signal at this position indicates that the ligation reaction takes place as expected. The scheme of the spotted zipcode oligonucleotides is shown in Figure 1.

**Figure 2:**
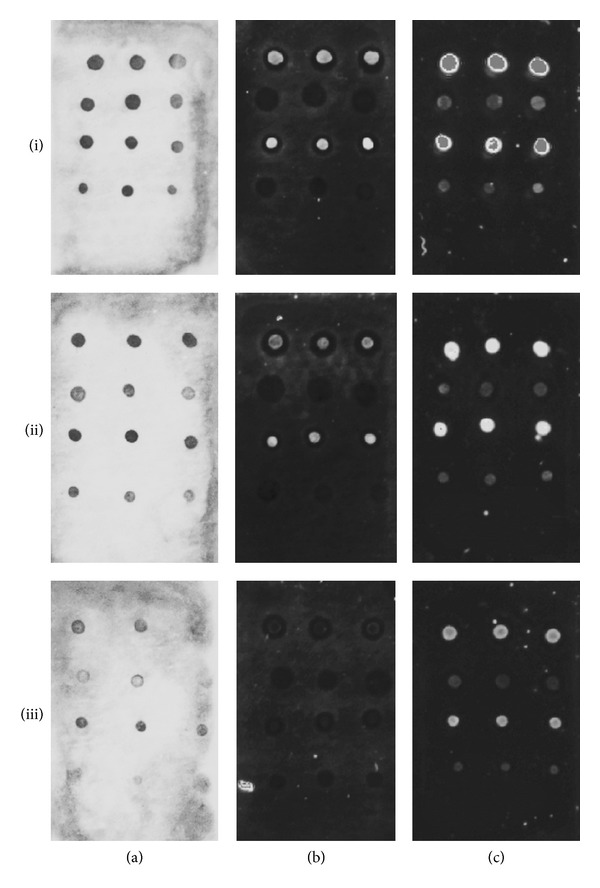
Biodyne C membrane-macroarray images obtained for optimization experiments with different labeling: (a) biotin, (b) Cy3, and (c) Cy5 and zipcode-oligonucleotides spotted at different concentrations: (i) 1 *μ*M, (ii) 0.1 *μ*M, and (iii) 0.01 *μ*M.

In these preliminary experiments, different labeling agents were tested (Figures 2(a)–2(c)) and different concentrations of the zipcode oligonucleotides were spotted onto the membranes ((i)–(iii) in Figure 2) to compare the intensities of the dots obtained after hybridization of the positive controls. Good results were obtained spotting the oligonucleotides at concentrations of 1 *μ*M and 0.1 *μ*M ((i) and (ii) in Figure 2), whereas the concentrations of 0.01 *μ*M gave dots with unacceptable levels of intensity. The best results were obtained with biotin labeling (Figure 2(a)) with good intensities for both hybridization and ligation controls. In the case of Cy3-labeling, no positive signal was detected for the ligation controls in any experiment (Figure 2(b)). This was due to a high fluorescence background all over the membrane that complicated the visibility of the spots. With Cy5-labeling little fluorescence background was obtained, but although the dots of the ligation controls could be clearly detected, the intensity was lower than that obtained with biotin labeling (Figure 2(c)).

In addition, different volumes of the spotted zipcodes were tested. Well-defined dots were obtained with volumes higher than 0.1 *μ*L, smaller volumes resulting to be less reproducible due to difficulties in pipetting. Nevertheless, smaller volumes and smaller dots could be obtained with adequate pipettes and tips and a macroarray with up to 50 spots could be designed.

**Figure 3:**
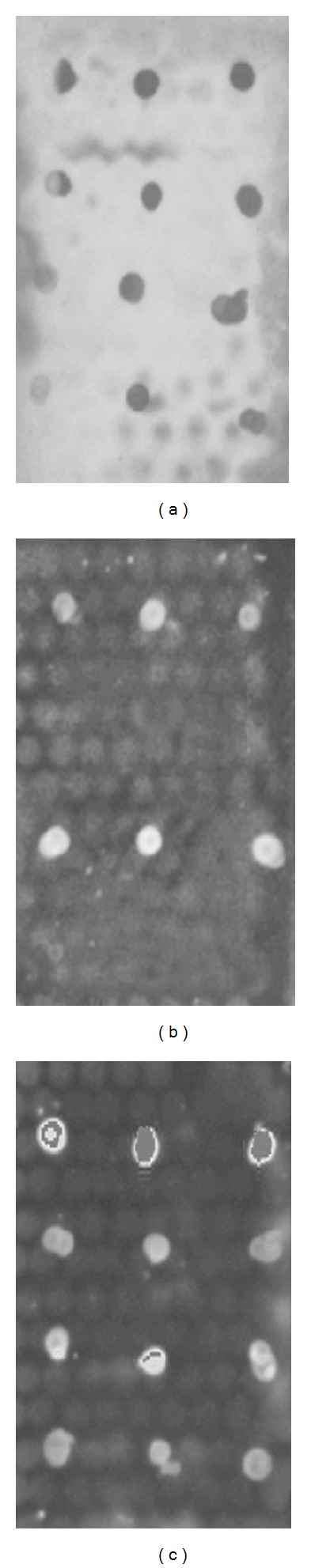
Nitrocellulose membrane-macroarray images obtained for optimization experiments with different labeling: (a) biotin, (b) Cy3, and (c) Cy5.

Biodyne C nylon membranes have been described to be the most adequate ones for hybridization purposes, since oligonucleotides are covalently bound onto the membranes by interaction between the negatively charged carboxyl groups of the membrane and the positively charged amino-groups of DNA. However, since nylon membranes are known to exhibit a higher fluorescence background compared to nitrocellulose membranes, the latter were also tested. In the case of nitrocellulose membranes, DNA oligonucleotides were directly spotted onto the membranes and cross-linked by UV-light. Unfortunately, the results were less satisfactory and the nitrocellulose membranes showed even a higher fluorescence background (Figure 3). Likewise, a high color background was observed for the biotin labeling. The higher background for all the three labeling reagents was mainly due to the difficulties in the washing processes, since the nitrocellulose membranes are less permeable. In addition, the spots obtained on nitrocellulose membranes were not as well defined as on nylon membranes.

**Figure 4:**
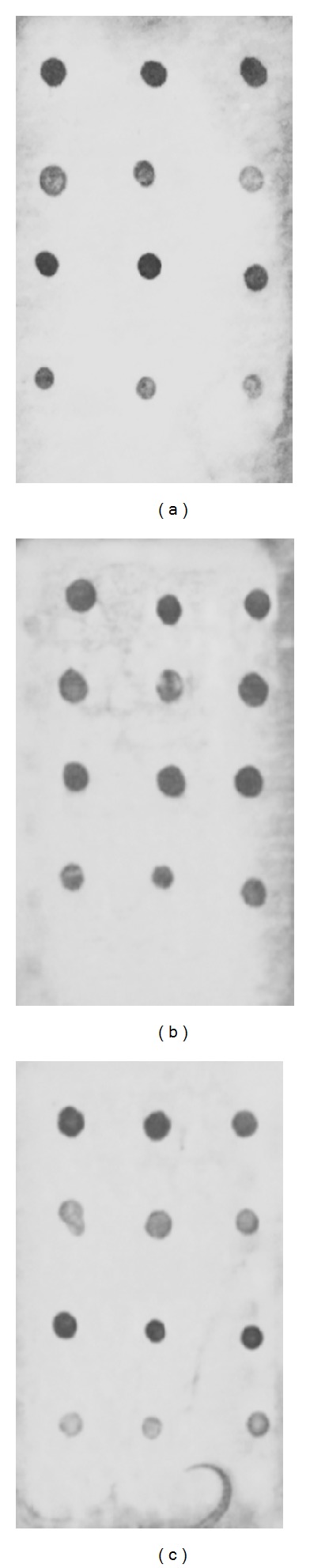
Biodyne C membrane-macroarray images obtained for optimization experiments with biotin labeling and different ligase enzymes: (a)* pfu* DNA ligase, (b)* taq* DNA ligase, and (c) ampligase.

In a further experiment, three different ligase enzymes were tested:* pfu* DNA ligase,* taq* DNA ligase, and ampligase. The best results were obtained with* taq* DNA ligase, exhibiting dots with higher intensities for the ligation controls.  Figure 4 shows the hybridization results on Biodyne C membranes and biotin labeling after carrying out the ligation reaction employing the three different enzymes. Similar results were obtained for the fluorescence label Cy5.

### 3.3. Application of the Designed LDR Probes to the Flow-Through Hybridization Methodology

**Figure 5:**
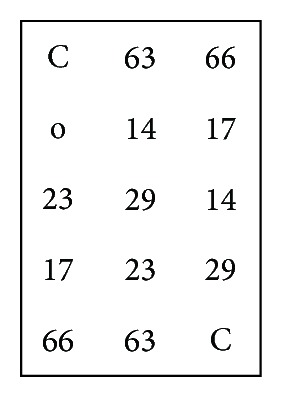
Scheme of spotted zipcode-oligonucleotides on membranes. ^C^Color control, ^66^hybridization control, ^63^ligation Control, ^14^ *Pseudomonas* spp., ^17^ *Aeromonas* spp., ^23^ *Shewanella* spp., and ^29^ *Morganella morganii*.

As a conclusion of the optimization experiments, the following application of the designed probes to the flow-through hybridization approach was carried out with* taq* DNA ligase in the ligation reaction and with biotin labeling for hybridization on Biodyne C membranes. The zipcode oligonucleotides were spotted at a concentration of 0.1 *μ*M and a volume of 0.2 *μ*L. The scheme of the spotted macroarray is shown in Figure 5.

**Figure 6:**
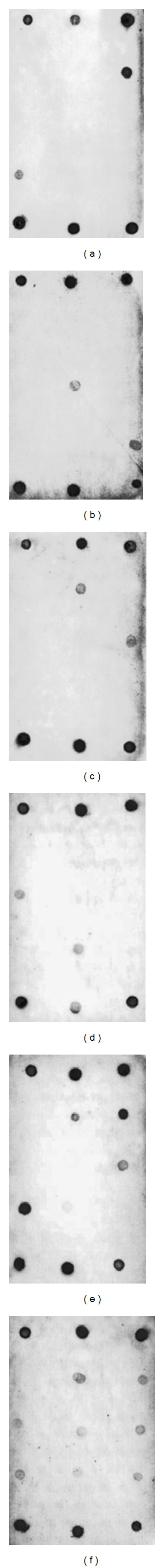
Biodyne C membrane-macroarray images obtained when testing the strains (a)* Aeromonas hydrophila* ATCC 7966, (b)* Morganella morganii* ATCC 8076, (c)* Pseudomonas fluorescens* ATCC 13525, (d)* Shewanella putrefaciens* ATCC 8071, (e) a mix of* A. hydrophila* ATCC 7966 and* P. fluorescens* ATCC 13525, and (f) a mix of all the four strains corresponding to the different species.

The methodology was validated with the strains listed in Table 2. With the flow-through hybridization approach bacterial DNA of the corresponding reference strains was successfully detected at concentrations of 10 fmol. Furthermore, the specificity of the designed probes was also confirmed with this technique, resulting in positive signals at the corresponding positions on the macroarray membrane (Figure 6) and no signal of the negative controls (*B. cereus* ATCC 14893 and* S. aureus* ATCC 9144) (data not shown). In addition, the methodology was also successfully applied for mixtures of amplified DNA from reference strains corresponding to different species (Figures 6(e) and 6(f)).

## 4. Discussion

Microarray-based methods represent state-of-the-art technologies for a high-throughput molecular analysis, allowing high sensitivity, accuracy, and specificity. DNA microarrays became a common tool in many areas of microbial research, including detection and identification of bacterial species, due to the high potential to discriminate subtle differences in the genome, the possibility to analyze multiple targets simultaneously, and the ability to detect noncultivable bacteria. In addition, a microarray experiment has the facility to be miniaturized and automated [1, 2, 18]. Thanks to the increase in the complete microbial genome sequencing and the advances in bioinformatics and biomolecular technologies, DNA microarrays are promising analytical tools that are expected to replace time-consuming and laborious conventional techniques for bacterial identification in near future. However, the impact on the food microbial sector has been less intensive and for food control purposes traditional culturing methods are still commonly applied due to the elevated costs and technical expertise required by microarray-based assays.

One of the challenges and greatest costs in the process of designing a microarray is the identification, testing, and validation of discriminatory gene regions [8]. Most designed oligonucleotide microarrays for bacterial identification are based on probes with the 16S rRNA gene as target sequence. Ribosomal genes represent ideal molecular markers for microbial identification by oligonucleotide arrays, first, because they contain conserved, as well as variable regions and second, due to the huge amount of sequence data available in public databases that facilitate the design of specific probes. Microarrays based on the 16S rRNA gene have been successfully applied for the detection of foodborne pathogens isolated from different food samples [3, 4] and coastal waters [5]. In another study, bacterial food pathogens, such as* Bacillus* spp.,* Escherichia coli*,* Salmonella* spp.,* Staphylococcus* spp., and* Vibrio* spp. were detected by a 16S rRNA based oligonucleotide array [19]. However, in this study the discrimination was achieved at the genus level but not at the species level.

The limitations of the 16S rRNA gene as a target are due to the high similarity of these gene sequences in many bacterial species. Nevertheless, discrimination of closely related bacterial species is often required, as well in clinical diseases as in the food safety sector, due to the varying pathogenic and spoilage character of genetically closely related species. In this sense, the 16S-23S rRNA intergenic spacer region (ISR) exhibits higher sequence variability, allowing the design of specific probes for the successful identification of bacterial foodborne pathogens [20–23]. In other studies aimed at the detection of foodborne pathogens, the designed probes referred to specific genes, such as virulence or toxin coding genes. This approach led to the unequivocal identification of a number of important foodborne pathogens, such as* Campylobacter jejuni*,* E. coli*,* Salmonella typhimurium*,* Listeria monocytogenes*, and* Yersinia enterocolitica* [24, 25]. However, the high specificity of this approach contrasts with the challenges of the need for multiplex amplifications before hybridization.

The methodology applied in the present study was based on a different microarray technology that combines the high specific ligase detection reaction (LDR) and the multiple, high-throughput DNA analysis of a microarray platform. The LDR approach has the ability to differentiate two DNA sequences by a single nucleotide polymorphism and requires the design of two oligonucleotide probes specific for each target sequence. In the ligase reaction both probes are hybridized to a present template and ligated only in the case of a perfect match in the discriminating position [26]. A further advantage of the LDR is that the ligation is carried out in liquid medium, allowing a much better interaction and hybridization of two complementary DNA strands that result in an increased sensitivity. Afterwards, the ligation products these including the joined and labeled probes hybridize to a certain location on a universal microarray. Since the ligated probes are short oligonucleotides, the hybridization process is facilitated considerably as compared to whole genes. In addition, the sequence fragments that hybridize to the array, namely, the zipcodes, are located at the end of the probes, again facilitating the contact to the immobilized DNA sequences. Such zipcodes are synthetic sequences that are unique and different from any existing sequence, thus avoiding cross-hybridizations. The number of zipcode sequences is unlimited and further probes can be designed at any time, assigning them to a “free” or new zipcode sequence that is spotted onto the universal microarray. Accordingly, the microarray platform can be enlarged easily for further species or genes and thus be applied to a wide range of microbial samples. This is of special interest for bacterial identification in the food sector, due to the fact that the universal microarray can be applied to any foodstuff, just adapting the mix of probes used for the ligation reaction by adding the probes that correspond to the target microbial species.

The application of LDR coupled to a universal array (UA-LDR) for microbial identification has been described by various authors [27–29], especially for clinical diagnostics. Cremonesi et al. [11] successfully applied the methodology to detect and identify bacterial species causing mastitis in dairy ruminants, also including species that are of great interest as responsible of foodborne disease and food spoilage.

The present study was based on one hand on the results obtained by these authors and, on the other hand, on the design of further probes using the same bioinformatics tool ORMA. Difficulties were observed for some genera, especially inside the Enterobacteriaceae family, for which species differentiation was not possible due to the high sequence similarity of the 16S rRNA gene and the great variety of closely related bacterial species that are potentially present in food products. Finally, four probes were selected and resulted to be specific for the genera* Aeromonas*,* Pseudomonas*, and* Shewanella*, as well as for the species* M. morganii*. The probes were successfully tested* in vitro* with the UA-LDR approach described by Cremonesi et al. [11], being specific and sensitive to concentrations of 5 fmol. This is the first time that LDR probes have been designed for these relevant foodborne bacterial species and genera.

As mentioned before, the UA-based approach can be easily enlarged by further probes corresponding to other bacterial species with food spoilage character, thus representing an efficient microarray platform with high potential for the accurate bacterial species identification in the food control sector, where a great variety of bacterial species can be present in one food product and need to be detected at low concentrations.

However, besides the fast technology development in the microarray field, the transition to potential commercial applications is very slow [8]. The main drawback of the microarray technology is the necessary large initial investment for sophisticated and expensive instruments needed for microarray printing and laser scanning. In addition, high technical expertise is required, thus limiting their applications to well-trained and well-equipped laboratories [9, 30].

In this sense, although the throughput of macroarrays is moderate as compared to microarrays, the former represent a cost-effective, rapid, accurate, and efficient alternative that do not require specialized equipment and can be used in any well set-up molecular laboratory but still retain the sensitivity and specificity of the microarray technology. Such oligonucleotide macroarrays are based on the reverse dot-blot (RDB) hybridization technique in which specific oligonucleotide probes are immobilized to membranes and afterwards the labeled target DNA is hybridized to the membranes [31, 32].

The application of a flow-through (FT) device for hybridization represents an improvement of the RDB technique. The FT-RDB approach has been described by various authors for specific oligonucleotide detection and resulted to be the most efficient method for molecular hybridization, combining two advanced techniques: flow-through hybridization and simultaneous multiple detection on a macroarray membrane. An active flow directs the sample DNA towards immobilized probes within the membrane fibers, increasing the diffusivity and local reaction concentration of DNA and allowing the close contact of DNA probes with the immobilized DNA inside the membrane pores. Conventional dot-blot hybridizations need to be incubated for several hours in plastic bags or glass tubes and require large volumes to cover the whole membrane, which decreases the concentration of DNA available for binding to the probes, also decreasing active interactions between complementary strands considerably [33]. The FT-RDB hybridization invention demonstrated to be an efficient approach for genotyping, being more efficient, faster (<15 min), and less expensive than gene chips, thus having high potential for the use in microbial identification.

The flow-through hybridization device HybriMax used in the present study has been patented by Tam [17]. Besides the advantages of flow-through hybridization mentioned above, with this device the hybridization process can be semiautomated, this being cleaner and less expensive than traditional hybridization protocols [17]. More recently, the HybriMax and FT-RDB hybridization process on nylon membranes were successfully applied to the genotyping of hepatitis B virus [34, 35]. The application of HybriMax to human papillomavirus (HPV) genotyping was also reported [36].

The aim of the present study was the use of the FT-RDB hybridization approach for the fast and efficient identification of food pathogens and spoilage bacteria. In another work, the combination of FT and RDB has been applied to the detection of ten intestinal foodborne pathogens (*Salmonella* spp.,* Brucella* spp.,* E. coli* O157:H7,* Clostridium botulinum*,* B. cereus*,* Clostridium perfringens*,* Vibrio parahaemolyticus*,* Shigella* spp.,* Y. enterocolitica*,* Vibrio cholerae*,* L. monocytogenes*, and* S. aureus*) [9]. In such study the bacterial 16S and 23S genes were simultaneously amplified directly from 540 fecal samples and the results were compared to traditional culture methods. Our study considered three probes designed by these authors. In this sense, the probe for* L. monocytogenes* exhibited cross-hybridization with* B. cereus* and* Bacillus thuringiensis* strains, while the probe for* S. aureus* cross-hybridized with* L. monocytogenes* strains. Likewise, the probe for* B. cereus* could not distinguish between* B. cereus* and* B. thuringiensis*, due to the high sequence similarity of the 16S and 23S rRNA genes of these two species.

In another study, RDB hybridization on nylon membranes was successfully applied for the detection of 14 foodborne pathogens, using specific probes for the 23S rRNA gene as target sequences for the species* E. coli*,* C. jejuni*,* Shigella dysenteriae*,* V. cholerae*,* V. parahaemolyticus*,* Proteus vulgaris*,* B. cereus*,* L. monocytogenes*,* C. botulinum*, and* S. aureus* [37]. However, these authors also reported problems of unspecificity during the hybridization process. Although the 23S rRNA gene exhibited more sequence variability than the 16S rRNA gene, the differentiation of closely related species is not easy. These authors described the difficulties in the design of specific probes for the species* C. perfringens* and* Streptococcus pyogenes* based on the 23S rRNA gene. Furthermore, the DNA isolated of the species* Salmonella enterica* and* Y. enterocolitica* gave cross-reactions with* E. coli* in their study [37].

In a recent study, 11 foodborne pathogens were detected with biotinylated species-specific primers and hybridization of the amplification products to specific probes on a thin-film biosensor [30]. The assay proved to be extremely robust, sensitive, specific, and economical, obtaining reliable PCR fragment identification within 30 min. However, the specificity of the sensor requires the amplification with species-specific primers, this involving multiplex PCR reaction, which limits the throughput and number of target species.

The assays described in the present work have been carried out taking into account the principles of FT-RDB hybridization described by Xing et al. [9], but resolving the above-mentioned problems by combining the approach with the high specific LDR probes. As a result, the HybriMax device was successfully applied for the detection and identification of bacterial reference strains by hybridizing the joined probes to immobilized DNA fragments on membranes, after carrying out PCR amplification and ligation reaction. The protocol has been optimized to improve the sensitivity of the method. In RDB hybridization experiments the results are normally visualized by biotin labeling and an enzyme-induced color development reaction. To avoid several washing and incubation steps that are required for this procedure, experiments have also been carried out with fluorescence labeling. However, instead of an expected higher sensitivity, the detected spots were less intense with fluorescence labeling, making biotin labeling and subsequent color development reaction the method of choice for the FT-RDB hybridization assay.

## 5. Conclusion

The FT-RDB hybridization methodology described in this work is fast and efficient and can be easily implemented in standard microbiological laboratories due to the ease of use and reduced costs as compared to the expensive instrumentation, material, and technical expertise required for microarray platforms. The assay can be applied for food control purposes, obtaining a rapid, accurate, and specific identification of bacterial species with importance in the spoilage and safety of any food product. In the PCR step, bacterial DNA could be amplified directly from a food matrix without previous isolation and purification steps. Thanks to the combination of specific LDR-probes and the flow-through hybridization process, results were obtained in a few hours. Food microbial risk can be efficiently achieved with the FT-RDB approach evaluated in this work, this resulting in a better quality control of foodstuffs and the prevention of foodborne disease.

## Figures and Tables

**Table 1:** Nucleotide sequences of the selected unique probes and complementary zipcodes.

Probe name^1^	Probe sequence^2^ czipcode sequence^3^	Pos.
Hybrid control	gttaccgctggtgctgccgccggta	66
Synth template	agccgcgaacaccacgatcgaccggcgcgcgcagctgcagcttgctcatg	
DS synth template	catgagcaagctgcagctgcgcgc**g**	
CP synth template	ccggtcgatcgtggtgttcgcggctgtggtgtgccagccgtcggtgccat	63
DS *Morganella *	ggcgtaaagcgcacgcaggcggttgatt**g**	
CP *Morganella *	agtcagatgtgaaatccccgggcttaacccgggatgtcagtgacgcgctcagcgttg	29
DS *Shewanella *	gatgtctactcggagtttggtgtcttgaacactggg**c**	
CP *Shewanella *	tctcaagctaacgcattaagtagaccgcctggggagccgtacccttccgctggagatttac	23
DS *Aeromonas *	gccccgggctcaacctgggaattgcatttaaaactg**t**	
CP *Aeromonas *	ccagctagagtcttgtagaggggggtagaattccagtgtgcgcccgagatcggtatccccg	17
DS *Pseudomonas *	cccttgtccttagttaccagcacgtiatggtggg**c**	
CP *Pseudomonas *	actctaaggagactgccggtgacaaaccggaggggattgcaccgtcagcaccaccgag	14

^1^DS: discriminating probe; CP: common probe; ^2^discriminating positions are indicated as bold; ^3^complemantary zipcode sequences are underlined.

**Table 2:** Reference strains used for probe validation.

Strain	
*Aeromonas caviae* ATCC 15468	
*Aeromonas hydrophila* ATCC 7966	
*Aeromonas veronii* ATCC 35624	
*Bacillus cereus* ATCC 14893^a^	
*Morganella morganii* BM-65	
*Morganella morganii* ATCC 8076	
*Pseudomonas fluorescens* ATCC 13525	
*Pseudomonas fluorescens *ATCC 17397	
*Pseudomonas fragi* ATCC 4973	
*Pseudomonas putida* ATCC 12633	
*Pseudomonas putida* ATCC 17453	
*Pseudomonas syringae* ATCC 19310	
*Shewanella algae* ATCC 51192	
*Shewanella baltica* CECT 323	
*Shewanella putrefaciens* ATCC 8071	
*Staphylococcus aureus* ATCC 9144^a^	

^a^Strains applied as negative controls.
